# Genetic Variations on Redox Control in Cardiometabolic Diseases: The Role of Nrf2

**DOI:** 10.3390/antiox11030507

**Published:** 2022-03-06

**Authors:** Cecilia Zazueta, Alexis Paulina Jimenez-Uribe, José Pedraza-Chaverri, Mabel Buelna-Chontal

**Affiliations:** 1 Departmento de Biomedicina Cardiovascular, Instituto Nacional de Cardiología, I.Ch., Mexico City 14080, Mexico; ana.zazueta@cardiologia.org.mx; 2 Departamento de Biología, Facultad de Química, Universidad Nacional Autónoma de México, Mexico City 04510, Mexico; jimenez.uribe.ap@comunidad.unam.mx (A.P.J.-U.); pedraza@unam.mx (J.P.-C.)

**Keywords:** redox control, Nrf2, single nucleotide polymorphisms, cardiometabolic diseases

## Abstract

The transcription factor Nrf2 is a master regulator of multiple cytoprotective genes that maintain redox homeostasis and exert anti-inflammatory functions. The Nrf2-Keap1 signaling pathway is a paramount target of many cardioprotective strategies, because redox homeostasis is essential in cardiovascular health. *Nrf2* gene variations, including single nucleotide polymorphisms (SNPs), are correlated with cardiometabolic diseases and drug responses. SNPs of *Nrf2*, *KEAP1,* and other related genes can impair the transcriptional activation or the activity of the resulting protein, exerting differential susceptibility to cardiometabolic disease progression and prevalence. Further understanding of the implications of *Nrf2* polymorphisms on basic cellular processes involved in cardiometabolic diseases progression and prevalence will be helpful to establish more accurate protective strategies. This review provides insight into the association between the polymorphisms of *Nrf2*-related genes with cardiometabolic diseases. We also briefly describe that SNPs of *Nrf2*-related genes are potential modifiers of the pharmacokinetics that contribute to the inter-individual variability.

## 1. Introduction

Worldwide cardiometabolic diseases-related deaths have been increasing over the last decades, representing a big concern [1]. As cardiometabolic diseases lead to unbalance of redox homeostasis and enhanced oxidative stress (for a recent comprehensive review, see [2]), different antioxidant therapies have been evaluated to prevent and treat such diseases. Although some of these have been designed to induce the nuclear factor erythroid 2-related factor 2 (Nrf2)-driven antioxidant response [3], their efficacy in the clinic has not been clearly established, giving rise to the possibility of the participation of other regulatory elements in the Nrf2-signaling cascade. In this regard, multiple single nucleotide polymorphisms (SNPs) have been found in the *Nrf2* gene [4]. Overall, SNPs of the *Nrf2* gene induce changes in Nrf2 activation, which can lead to the impairment of the endogenous antioxidant system, contributing to chronic inflammation and other detrimental effects [5]. Indeed, several polymorphisms of *Nrf2* have been associated with cardiometabolic diseases progression [6]. This review highlights the association of SNPs of *Nrf2* and related genes with cardiometabolic diseases that significantly impact disease progression, as well as a short look at the possible implications on cardiovascular drug therapy.

## 2. The Role of Nrf2 to Maintain Redox Homeostasis in Cardiometabolic Diseases

A common feature of cardiometabolic diseases is the imbalance between pro- and anti-oxidative factors in the cell. Such condition is associated with high levels of reactive oxygen species (ROS) and reactive nitrogen species (RNS) that react with lipids, proteins, DNA, or activate redox signaling pathways, leading to cellular injury and death. Disruption of the redox homeostasis is associated with poor cardiac contractility related to reduced sarco-endoplasmic reticulum Ca^2+^-ATPase (SERCA2A) activity [7], but also with mitochondrial damage. Mitochondria are the primary ROS producers and, at the same time, are the first exposed in front line due to their deleterious action, resulting in further mitochondrial dysfunction and a vicious cycle of ROS generation. Impairment of the mitochondrial function and increased rates of fatty acid β-oxidation promote the accumulation of incomplete β-oxidation products, which, in combination with oxidative stress, contribute to insulin resistance in cardiometabolic diseases [8].

**Figure 1:**
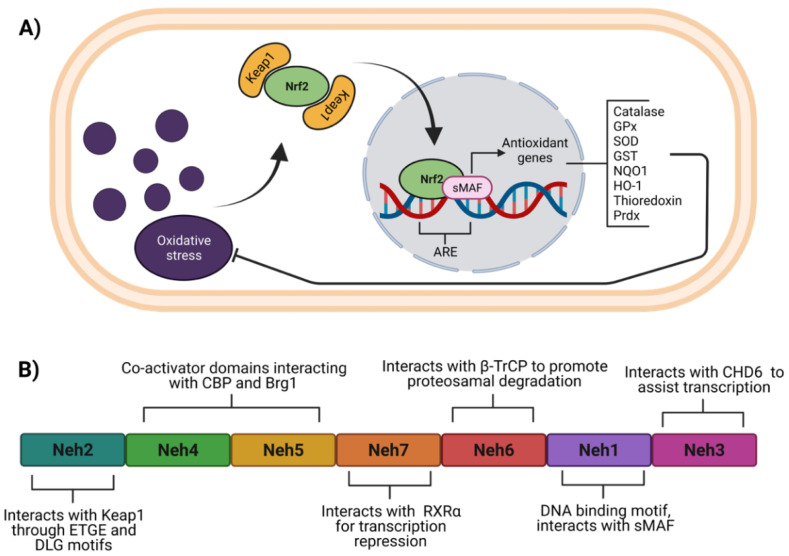
Function and structure of Nrf2. (**A**) Under oxidative stress, Nrf2 dissociates from Keap1, to be translocated to the nucleus where heterodimerizes with the sMAF proteins for recognition of ARE within several genes to induce their expression. (**B**) Nrf2 consists of seven domains named Neh1 to 7, which allow specific interaction for its regulation. ARE, antioxidant response elements; β-TrCP, beta-transducin repeat-containing protein; Brg1, Brahma-related gene 1; CBP, CREB binding protein; CDH6, chromo-ATPase/helicase DNA binding protein 6; GPx, glutathione peroxidase; GST, glutathione-S-transferase (GST); HO-1, heme-oxygenase 1; Keap1, Kelch-like ECH-associated protein 1; NQO1, NAD(P)H quinone oxidoreductase 1; Neh, Nrf2-ECH homology; Nrf2, nuclear factor erythroid 2-related factor 2; Prdx, peroxiredoxins; RXRα, retinoid X receptor alpha; SOD, superoxide dismutase; sMAF, small musculoaponeurotic fibrosarcoma. Figure created with BioRender at biorender.com (accessed on 10 February 2022).

In cardiometabolic diseases, among the antioxidant molecules regulated by Nrf2 are catalase (CAT), superoxide dismutase (SOD), glutathione S-transferases (GST), glutathione peroxidases (GPx), heme-oxygenase 1 (HMOX-1), thioredoxin (TXN), thioredoxin reductases, peroxiredoxins (Prdx), and NAD(P)H quinone oxidoreductase-1 (NQO1) [9,10,11,12] (Figure 1A). Besides the antioxidant response regulation, Nrf2 also modulates a myriad of genes related to other cellular processes. For instance, it is known that Nrf2 regulates the expression of Notch1 and downstream genes, which collectively coordinate signals that affect differentiation, proliferation, and apoptotic events [13]. Moreover, it was demonstrated by chromatin immunoprecipitation that Nrf2 binds directly to an antioxidant response elements (ARE) region in the aryl hydrocarbon receptor (AHR) promoter [14]. AHR increased in pig myocardium after ischemia-reperfusion damage and diminished upon administration of natural flavone baicalin [15] and sulforaphane [16] in association with lesser cardiac injury and inflammation.

### Nrf2 Structure and Regulatory Mechanisms

Nrf2 belongs to the cap‘n’ collar (CNC) subfamily of the basic leucine zipper (bZip) transcription factors [17]. Nrf2 contains seven domains (Figure 1B) named Nrf2-ECH homology (Neh) 1 to 7 [18,19]. Among Neh domains, Neh1 and Neh2 have the most recognizable roles in Nrf2 function and regulation. Neh2 domain is located at the amino-terminal and has ETGE and DLG motifs that interact with Kelch-like ECH-associated protein 1 (Keap1), the well-known negative regulator of Nrf2 [20]. Neh4 and Neh5 are two domains that allow Nrf2 interaction with other proteins such as co-activators [21,22,23,24], whereas Neh5 possesses a nuclear export signal (NES) sensitive to oxidation [25]. Neh7 and Neh6 domains act as negative regulators, and Neh7 interacts with the retinoic X receptor alpha (RXRα) to repress Nrf2 transcriptional activity. In fact, RXRα competes with Nrf2 for binding to the ARE sequence [19]. On the other hand, Neh6 interacts with beta-transducin repeat-containing protein (β-TrCP) to promote Nrf2 ubiquitination and consequent degradation [26,27]. Neh1 domain located near the C-terminal region harbors a basic leucine zipper motif, which binds to ARE sequences within target genes. Neh1 also interacts with small musculoaponeurotic fibrosarcoma (sMAF) proteins favoring the transcription of antioxidant genes. In addition, Neh1 acetylation enhances the transcriptional activity of Nrf2 [28,29]. The Neh3 domain in the C-terminal region interacts with chromo-ATPase/helicase DNA binding protein 6 (CDH6), essential for Nrf2 transcriptional activity [30].

**Figure 2:**
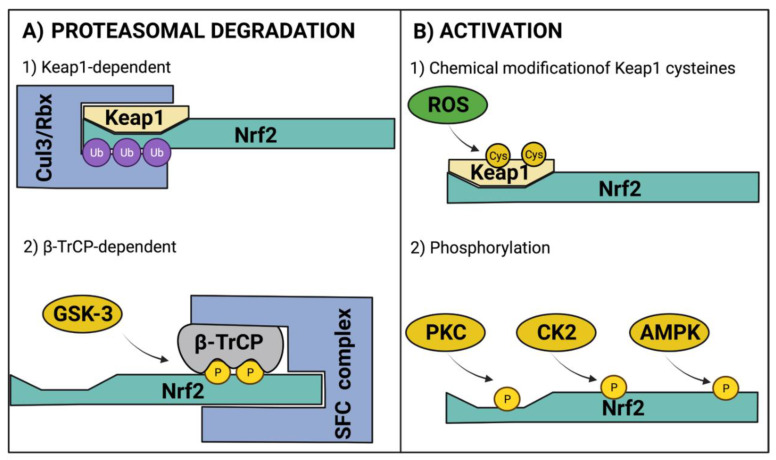
Regulatory mechanisms of Nrf2. (**A**) For Nrf2 regulation at protein level, Nrf2 is ubiquitinated (Ub) for its proteasomal degradation through Keap1-dependent and β-TrCP-dependent mechanisms. (**B**) For Nrf2 activation, the canonical pathway involves the chemical modification of Keap1 cysteines (Cys) that elicits the release of Nrf2 for its nuclear translocation. Additionally, phosphorylation (P) by cytosolic kinases promotes Nrf2 activation. AMPK, AMP-activated protein kinase; β-TrCP, beta-transducin repeat-containing protein; CK2, casein kinase 2; Cul3, cullin 3; GSK-3, glycogen synthase kinase 3; Keap1, kelch-like ECH-associated protein 1; Nrf2, nuclear factor erythroid 2-related factor 2; PKC, protein kinase C; Rbx, ring box-1; ROS, reactive oxygen species; SFC, Skp1–Cullin–F-box protein complex. Figure created with BioRender at biorender.com (accessed on 10 February 2022).

Under homeostatic conditions, Nrf2 is maintained at low levels due to constant proteasomal degradation. The degradation process occurs through the interaction of the ETGE and DLG motifs within the Nrf2 Neh2 domain with a Keap1 homodimer [20]. Keap1 acts as an adaptor for an E3 ubiquitin ligase complex that contains cullin 3 (CUL3) and ring box-1 (Rbx) [31], which catalyze the ubiquitination of lysine residues located between ETGE and DLG motifs [32,33] to induce Nrf2 degradation through the ubiquitin proteasomal system (UPS). Interestingly, Nrf2 also promotes CUL3 and Rbx expression as negative feedback [34]. Another mechanism that induces Nrf2 degradation via UPS is Neh6 phosphorylation by glycogen synthase kinase 3 (GSK-3); this promotes the binding of β-TrCP, which acts as a substrate for the Skp1–Cullin–F-box protein complex (SFC), another E3 ubiquitin ligase complex (Figure 2A) [35]. Of note, the levels of GSK-3 increase in experimental models of obesity, diabetes, and associated cardiovascular diseases [36,37,38].

During oxidative stress, ROS can react with Keap1 cysteine residues modifying the Keap1 homodimer structure, allowing Nrf2 release. Hence, Keap1 also acts as a sensor of redox homeostasis [39,40,41,42,43]. Once Nrf2 is released from the Keap1/Cul3/Rbx complex, nuclear localization signals (NLS) are exposed for importins recognition to elicit Nrf2 nuclear translocation [44]. After heterodimerization with sMAF, Nrf2 induces the expression of antioxidant response-related genes [45]. Another potential mechanism for Nrf2 activation involves its phosphorylation by several kinases. Protein kinase C (PKC) phosphorylates Nrf2 at serine 40 of the Neh2 domain, inducing its nuclear translocation [46]. Casein kinase 2 (CK2) can phosphorylate Neh4 and Neh5 transactivation domains, enhancing Nrf2 transcriptional activity [47]; in addition, CK2 phosphorylates AMP-activated protein kinase (AMPK) [48], which, in turn, phosphorylate serine residue 558 to increase Nrf2 nuclear accumulation [49], and serine residues 374, 408, 433 to increase its transcriptional activity [50]. The regulatory mechanisms of Nrf2 are depicted in Figure 2.

## 3. *Nrf2* Genetic Variations (Single Nucleotide Polymorphisms)

Human *Nrf2* gene (NFE2L2 gene ID: 4780) codes for Nrf2 mRNA 2859 base pairs long, which is translated into a protein containing 605 amino acid residues. Genome-wide association studies (GWAS) have been used to find sequence mutations of the *Nrf2* locus including exonic SNPs [6]. Certainly, SNPs of the promoter region of the *Nrf2* and ARE-related genes regulate cardiometabolic disease progression, as we will detail later. To date, multiple SNPs of the *Nrf2* human gene have been found [4,6], including −617 C/A (rs6721961) and −651 G/A (rs6706649) in the promoter region, which attenuate Nrf2 transcriptional activity, diminishing ARE binding [51,52]. Likewise, the polymorphisms −686 A/G, −684 G/A, and −650 C/A and one triplet repeat polymorphism, −20 to −6 (CCG) 4 or 5, alter the basal expression of *Nrf2*, resulting in the increased severity of implicated diseases [5]. In the setting of chronic kidney disease, *Nrf2* (−617 C/A) rs6721961, *MnSOD* (*SOD2*) rs4880, and *GPx1* rs1050450 polymorphisms have been described as predictors of overall survival. Both rs6721961 and rs4880 impair antioxidant defense affecting survival, whereas rs1050450 contributes to longer cardiovascular survival in patients undergoing hemodialysis [53]. Furthermore, the polymorphism −653 A/G (rs35652124) in the *Nrf2* gene was related to a higher inflammatory profile in liver samples from alcoholic liver disease patients [54]. Additionally, the *Nrf2* rs35652124 and *KEAP1* rs1048290 variants have been associated with chronic inflammatory diseases such as chronic obstructive pulmonary disease [55]. In this context, genetic variations in *KEAP1* rs11085735 and *Nrf2* rs2364723 can affect lung function [56]; in particular, the *KEAP1* rs11085735 variant has been proposed as an independent strong predictor for incidence of cardiovascular events [57]. Furthermore, the functional polymorphism in promoter of *Nrf2* gene (−617 C/A) is associated with a higher risk to develop oxidative stress-related and inflammatory illnesses, including acute lung injury [58]. An interesting 18-year follow-up study in 1390 subjects from the Vlagtwedde–Vlaardingen cohort, a general Caucasian population-based cohort, demonstrated that *Nrf2* is associated with human survival. Carriers of the minor allele rs13001694 had less risk of all-cause mortality, whereas those carriers of rs2364723 showed a reduced risk for cardiovascular mortality and lower triglyceride levels [59]. 

Polymorphisms in the *Nrf2* gene and related genes that decrease Nrf2 activation might be a risk for several diseases. Additionally, human aging is a detrimental condition, where Nrf2 activation declines due to genetic variations. Elder patients carrying the rs35652124 variant in the *Nrf2* gene show frailty, adverse drug reactions, and multimorbidity [60]. The decreased Nrf2 activation in aging concurs with unbalance of redox homeostasis, supporting the theory that oxidative stress and the proinflammatory state contribute to aging and to an increase in cardiovascular diseases. Hence, either genetic or non-genetic factors leading to the impairment of Nrf2 transcriptional activity, including Nrf2 de novo synthesis, not only cause a faulty endogenous antioxidant system but also contribute to the development of several chronic inflammatory diseases [54].

## 4. Contribution of *Nrf2/Keap1* and Target Genes Polymorphisms in Cardiometabolic Diseases

### 4.1. Obesity and Diabetes

Obesity is defined as an imbalance between energy intake and energy expenditure resulting in adipose tissue hyperplasia; however, this pathology involves a complex network of metabolic alterations at cellular, tissular, and systemic levels that lead to low-grade inflammation and oxidative stress. In addition, this pathology is the hub for developing other non-communicable pathologies such as type 2 diabetes mellitus (T2DM), hypertension, steatohepatitis, and metabolic syndrome. Recently, obesity has been described as a low-grade state of chronic inflammation that also drives to an oxidative stress status in different organs besides adipose tissue; it is well known that both inflammation and oxidative stress can promote insulin resistance and T2DM development [61]. Redox imbalance occurs in obese [62,63,64,65] and in diabetic patients [66]; particularly in T2DM, oxidative stress is potentiated due to the presence of other inducers such as advanced glycation products derived from chronic hyperglycemia [67]. Hence, a great variety of natural Nrf2 activators has beneficial effects in experimental models of obesity and T2DM, showing a reduction in the inflammatory response, decrease in body weight gain, and lesser clinical complications [68,69,70,71,72,73,74,75,76,77]. The abovementioned supports the crucial role of Nrf2 in preventing the metabolic derangements associated with obesity and T2DM. Lower Nrf2 activity in experimental models of insulin resistance and diabetes has been found in different organs [78,79]. In addition, genetic variation contributes to Nrf2 dysfunction in obesity and in T2DM development, as we will detail further. 

*Nrf2* polymorphism rs2364723 has been correlated with changes in heart rate variability through an imbalance of autonomous nervous system in overweighted patients (SAPALDIA cohort), impairing cardiovascular function [80]. Moreover, polymorphisms found in *Nrf2*-related genes including *SOD1* (rs2234694), *SOD3* (rs2536512), *GSTM1* (rs1056806), *SOD2* (rs4880), and *GPx1* (rs1800668) were prevalent in obese patients, and the last four were associated with increased risk of obesity [81]. Another study found a positive association between elderly obese subjects and the *SOD2* rs4880 polymorphism, independent of other factors [82]. It was suggested that this polymorphism attenuates SOD2 activity and increases oxidative stress [83]. In addition, rs4880 was associated with higher oxidized-LDL (ox-LDL) levels and T2DM prevalence [84]. In this regard, a study in Japanese–American subjects indicates that *SOD2* rs4880 polymorphism might be associated with the progression of T2DM supported by observed oxidative stress, which leads to glucose intolerance [85]. On the other hand, attenuated SOD2 activity in those patients carrying the rs4880 polymorphism affects not only redox homeostasis but also increases the cardiovascular risk associated with T2DM and inflammatory response related to the ox-LDL, which contributes to the onset and progression of atherosclerosis [84]. Although the valine/alanine polymorphism of *SOD2* (rs4880) was not associated with diabetes etiology, it was associated with diabetic nephropathy in Japanese patients with T2DM [86]. Moreover, the rs4880 variant increased the risk for diabetic nephropathy in type 1 diabetes patients [87,88] and is a predictor of cardiovascular disease [88]. The *HMOX1* gene is associated negatively with diabetic complications, since the rs2364723 CG or CC allele increased in serum of T2DM patients compared to controls [89]. Additionally, the *GPx* rs1050450 has been associated with morbid obesity development in Mexican population, particularly in females [90]. This polymorphism is also related with diabetic neuropathy in a Polish and English population [91,92], as well as with the development of carotid plaques in a Chinese population with diabetes [93].

Among the polymorphisms on the *Nrf2* gene, seven are pointed out as relevant in T2DM and related complications. The polymorphism rs6721961 in the promoter region of the *Nrf2* gene was associated with T2DM development in Mexican patients [94], as well as diabetic complications such as diabetic nephropathy in Chinese patients [95]. Indeed, rs6721961 was strongly associated with a higher risk for the progression of T2DM in a Chinese population. The authors proposed that this polymorphism may impair β-cell function and increase insulin resistance, contributing to the onset and progression of T2DM [96]. Furthermore, the rs35652124 polymorphism, also located at the *Nrf2* promoter region, seems to be a harmful genetic variant that predisposes subjects to insulin resistance and impaired angiogenesis, associated with diabetic foot ulcer development, in an Indian population [97]. The *Nrf2* rs182428269 variant has been associated with T2DM risk and diabetic-associated complications in the Indian population [98]. On the other hand, the *Nrf2* polymorphisms rs2364723, rs10497511, rs1962142, and rs6726395 have not been associated with the risk of developing T2DM but were significantly associated with diabetic complications in Chinese patients [89]. A recent study suggests that Nrf2 expression-associated variants are likely to be a useful indicator of T2DM development in the human population [99]. 

The above reported suggest that *Nrf2* and -related genes polymorphisms are associated with obesity development, diabetes prevalence and progression, as well as diabetes-related complications, highlighting the role of redox homeostasis in diabetes pathophysiology.

### 4.2. Coronary Artery Disease (CAD)

CAD is a multifactorial chronic disease associated with several risk factors such as hypertension, diabetes, obesity, dyslipidemia, smoking, and genetics. The major cause of CAD is atherosclerosis, which results from the complex interplay between dyslipidemia, endothelial dysfunction, and oxidative stress. In the last decade, it has become evident the relationship between reduced Nrf2 activity and the development of cardiovascular disorders in patients with obesity, diabetes mellitus, and atherosclerosis [100,101], confirming a plethora of studies using pathological models in isolated cells as well as in animals, where the low activity of the Nrf2-Keap1-ARE axis correlates with augmented oxidative stress and detrimental cellular effects. The regulatory mechanisms of these pathways have been reviewed in depth [102,103]. However, the information about the genetic variability on this pathway among individuals, groups, or populations is still scarce. 

The survival analysis of the Vlagtwedde–Vlaardingen cohort, a general population-based cohort of Caucasian individuals of Dutch descent recruited from both a rural area and an urban area in the Netherlands, supports the hypothesis that *Nrf2* may be a gene that contributes to individual differences in human lifespan. The A/G substitution in the rs13001694 variant may occur only under oxidative stress conditions (smoking habits) and was associated with a lower risk of all-cause mortality. Those minor allele carriers of rs2364723 are associated with a reduced risk of cardiovascular mortality within groups with different risk factors, i.e., gender and smoking. The rs2364723 prevalence was associated with lower triglyceride levels in male subjects, supporting that this *Nrf2* variant contributes to the lower cardiovascular mortality observed [59]. Conversely, the rs35652124 polymorphism was associated with cardiovascular mortality in Japanese patients subjected to hemodialysis. A controversial issue in this study was that the G allele carriers of the rs35652124 variant, which is, in turn, associated with a diminished Nrf2 function, showed low cardiovascular mortality [104]. A possible explanation is that such an effect occurs through Nrf2 regulation on plasma lipoproteins and cholesterol, which overwhelms its antioxidant function. Studies in *Nrf2* knock-out mice exhibited greater oxidative stress and concurred with minor aortic atherosclerosis, sustaining such a hypothesis [105]. Some of the mechanisms involved include decreased CD36 expression, and thereby decreased LDL uptake and reduced cholesterol-dependent inflammasome activation [106]. Recently, using two different murine models of atherosclerosis, LDL receptor-deficient mice (*LDLR*^−^^/−^) and *LDLR*^−^^/−^ mice expressing only apoB-100 (LDLR^−^^/−^ApoB100/100), demonstrated that *Nrf2* deficiency delays early atherogenesis but, in later stages of the disease, promotes plaque instability [107]. Additionally, in a study that included 2374 Thai subjects with a high risk of CAD and without CAD, the association between *Nrf2* rs6721961 TT, the severity of coronary atherosclerosis, and CAD was demonstrated in the entire population, whereas the association with the *NQO1* rs1800566 CC genotype was established only in female [108]. 

Other studies have focused on studying variations in genes involved in oxidative stress at early atherosclerosis. For instance, *p66Shc* is an *Nrf2*-regulated gene [109] whose deletion correlated with lower oxidative stress, as well as reduced early atherogenic lesions in knockout *p66Shc*^−^^/−^ mice under a high-fat diet [110]. The *p66Shc* genetic variations, −354T>C and 92C>T, are scarce in subjects at the early onset of coronary artery disease, although these gene variants are not associated with the risk for CAD [111]. On the other hand, due to the risk of coronary events in chronic kidney disease patients being comparable with those with diabetes, the association of the genetic variability of the Nrf2-Keap1 axis in cardiovascular diseases has also been reported in those patients. In chronic kidney disease patients, a cohort carrying the A allele in the rs11085735 polymorphism located in the proximity of exon 3 of *KEAP1* was defined as an independent predictor of incident cardiovascular events [57].

Studies of SNPs of genes that contain ARE sequence in their promoters are scarce. Data from integrated computational analysis, chromatin immunoprecipitation sequencing (ChIP-Seq), and GWAS have been fundamental to identify some functional SNPs of ARE-containing genes [112]. Wang et al. [113] reported that the polymorphic ARE rs242561 C/T in *MAPT* gene (encoding microtubule-associated protein Tau) showed a strong affinity for Nrf2/sMAF heterodimers [113], likely inducing diverse responses in diseases in which conditions of oxidative stress prevail. Another example is the SNP rs113067944 within the ARE sequence of the ferritin light polypeptide (*FTL*) gene promoter. Changes in allele A to C in the core ARE region TG[A/C]CTCAGC decreased the Nrf2 binding affinity, resulting in a lower *FTL* transcription. In the study conveyed by Kuosmanen and coworkers [114], 14 different SNPs of the ARE sequences with putative clinical relevance have been described. Cardiovascular diseases were not considered in this analysis; however, it is well known that genes implicated in the metabolism of intracellular iron and ferroptosis, including ferritin light chain 1, are associated with cardiomyopathy and related diseases, such as myocardial infarction [115]. Conversely, the AA genotype of *HMOX1* was associated with the diminished incidence of ischemic heart disease in a Japanese study that included 1972 control subjects and 597 patients with myocardial infarction [116]. On the other hand, the role of the rs1800566 T carrier genotype (CT + TT) in the *NQO1* gene is controversial. Whereas Han et al. [117] have established an association with a higher risk to develop carotid artery plaques, other groups have reported that *NQO1* rs1800566 (CT + TT) has a protective effect on CAD risk [118]. Of note, there is a clear correlation between allele dosage and enzymatic activity in NQO1 rs1800566, i.e., the homozygotes (TT) have the lowest, the heterozygotes (CT) intermediate, and the wild-type homozygotes (CC) the highest NQO1 enzyme activity [119]. Interestingly, an association was found between the Val16Ala-SOD2 polymorphism of the *SOD2* gene and oxidative stress injury in hypercholesterolemic patients [120]. We need a better comprehension of how SNPs of the *Nrf2* and target genes contribute to the occurrence and progression of CAD to perform efficient redox-targeted therapy to treat cardiovascular diseases.

### 4.3. Hypertension

Nrf2-Keap1-ARE axis is fundamental in the antioxidant defense response in cardiovascular diseases, including atherosclerosis and hypertension [121]. Polymorphisms of genes coding for antioxidant regulators and enzymes might contribute to the modulation of their function in both conditions. As we mentioned before, polymorphisms of the promoter region of the Nrf2 gene might cause lower transcription, activity, and related gene expression. One of these SNPs, the rs6721961 (G>T) variation, was associated with elevated systolic and diastolic blood pressure levels and with cardiovascular mortality in Japanese patients undergoing hemodialysis, especially in the female cohort [104]. This finding was later confirmed by Kunnas et al. [122] in the TAMRISK study, in which hypertensive subjects were compared with normotensive ones in a Finnish cohort of 50-year-old subjects, followed up for ten years. They demonstrated that rs6706649 polymorphism located in the 5′ regulatory region of Nrf2 was not associated with cardiovascular disease. Endothelial dysfunction is a well-known factor implicated in hypertension. In this regard, individuals carrying the Nrf2 polymorphisms -617A and -653G showed impaired forearm vasodilator responses, affecting their vascular function [123].

HMOX1 gene has at least two polymorphic sites in its promoter at the 5′-flanking region, the rs3074372 (GT)n dinucleotide repeat, and rs2071746 T/A [124]. The A allele has been associated with HMOX1 higher transcription rates and higher blood pressure in women [116]. Moreover, the SOD3 c.172G>A variant and CAT c.-20C>T influenced blood pressure values and were associated with the risk for hypertension. Likewise, the polymorphisms of TXN gene c.-793T>C and GPx1 gene c.*891C>T also influenced blood pressure values [125]. Finally, the NQO1 rs1800566 CC genotype and HMOX1 rs2071746 showed marginal association with hypertension that increased when the combined effect was explored [108]. Therefore, this suggests that different genetic variants might interact or have synergic effects, resulting in increased susceptibility for CAD development risk factors. The SNPs of Nrf2 and related genes associated with cardiometabolic diseases are shown in Table 1.

## 5. Implications of *Nrf2*-Related Genes Polymorphisms in Cardiovascular Drug Therapy

Genetic polymorphisms cause interindividual variability that impacts the pharmacokinetics of anti-platelet, anti-coagulant, statins, and other drugs used for cardiovascular treatment, impairing their efficacy. Here, we briefly focus on some genetic polymorphisms of antioxidant-related genes.

Human UGT (UDP-glucuronosyltransferase)-2B7 regulated transcriptionally by Nrf2 is crucial for the metabolism of several drugs. Among other compounds, UGT drives phase II glucuronidation of the anticoagulant warfarin and its metabolite, hydroxywarfarin. Stable doses of warfarin in patients with mechanical cardiac valves were different among genotypes. In particular, the patients with the *UGT1A1* (rs887829) variant required higher doses of warfarin [126]. Genetic polymorphisms of the *UGT2B7* promoter region impair Nrf2-mediated transcription altering the drug efficacy [127]. On the other hand, the antiplatelet clopidogrel requires cytochrome P540 enzymes (CYPs) for its biotransformation into thiol active metabolites. Genetic variations of *CYP2C19* (*2 and *3 alleles) exert inter-individual variability in the pharmacokinetics of clopidogrel dosing [128]. Conversely, in patients with acute coronary syndrome, the polymorphism Q192R of paraoxonase-1 enzyme (*PON1*), which codes for a high-density lipoprotein-associated antioxidant enzyme (containing ARE/Nrf2 binding sites in its promoter), did not hamper the biotransformation of clopidogrel, unaltering its potential efficacy to treat cardiovascular events [129]. A common cardiovascular treatment is the use of statins to lower circulating total cholesterol (TC) and low-density lipoprotein cholesterol (LDL-C). Rosuvastatin is a statin used in clinical trials for LDL-cholesterol lowering, which also decreases ROS production, inflammation, and induces an upregulation of antioxidant enzymes [83]. Therefore, rosuvastatin maintains both redox homeostasis and vascular function, preventing atherogenesis. Moreover, hypercholesterolemic patients carrying the VV genotype of the Val16Ala-SOD2 polymorphism showed low SOD2 activity and resistance to rosuvastatin therapy, with a concomitant increase in inflammatory and fibrinolytic biomarkers [83]. On the other hand, the *CAT* gene polymorphism CAT-262C>T in dyslipidemic patients was not associated with insulin resistance, blood lipids increase, or response to atorvastatin [130]. Recently, polymorphisms of *SELENOP* (selenoprotein P), rs3877899, and rs7579 were associated with differential responses to selenium, whose accumulation alters redox homeostasis and lipid profile in patients after Brazil nut consumption and statins treatment [131].

Based on this evidence, it is clear that inter-individual variation defined by genetic polymorphisms may determine drug efficacy and particular doses for cardiometabolic diseases’ treatment.

## 6. Coffee Consumption, Genetic Polymorphisms of *Nrf2* and Cardiometabolic Diseases

We will briefly analyze the influence of coffee consumption on the risk of developing cardiometabolic diseases and its relation to polymorphisms of *Nrf2*-related genes. Coffee consumption has shown to be beneficial against cardiometabolic diseases. The acute effects of caffeine are increased metabolic rate/thermogenesis, insulin secretion, and reduced mean arterial pressure [132]. Indeed, moderate coffee intake in patients regulates lipid metabolism and prevents lipid oxidation and inflammation [133]. Recently, a meta-analysis discarded the idea that coffee or caffeine consumption is associated with the risk of atrial fibrillation [134]. Moderate coffee drinking is associated with a low risk of developing cardiometabolic diseases, such as T2DM [135], obesity [136,137,138], and heart failure [139]. Coffee-related beneficial effects may be mediated by coffee phytochemicals through an adaptive mechanism involving AHR, AMPK, sirtuins, and Nrf2-driven antioxidant genes transcription [135]. Conversely, high doses of caffeine can be detrimental to cardiovascular health by increasing blood pressure and heart rate [132]. Additionally, long-term high coffee consumption can increase serum triglycerides levels, total, and LDL-cholesterol, likely due to cafestol (a diterpene found in coffee), thus augmenting the risk for cardiometabolic diseases [140]. Certainly, the detrimental effects related to high coffee consumption involve a higher risk of CAD [141]. However, it has also been claimed that high coffee consumption is associated with lower risk for cardiovascular mortality [142]. These apparent contradictions might be associated with several factors, including genetic variability. On the other hand, coffee harvesters carrying the *PON1* Q192R variant showed decreased PON1 activity, affecting the hydrolysis of pesticides and oxidized lipids, which caused increased intoxication and higher cardiovascular risk, associated with hypertension [143]. Accordingly, high PON1 enzymatic activity is associated with a lower cardiovascular risk [144].

In this regard, coffee intake induces Nrf2-dependent gene expression, although, depending on prevailing SNPs of the *Nrf2* gene, high individual variability in the antioxidant response occurs [51]. A study showed that after four weeks of coffee intake, *Nrf2* gene transcription increased in individuals carrying the SNPs rs6721961 (-617 C/A), rs35652124 (-653 A/G), and rs6706649 (651 G/A) [52]. Similarly, a two-month coffee consumption trial showed that in rs35652124 carriers, *Nrf2*, *GST1A1*, and *UGT1A1* gene transcription increased compared to those carrying the rs6706649 genotype. Interestingly, before coffee consumption, the polymorphism rs35652124 was associated with lower Nrf2 transcription and further activation, whereas rs6706649 carriers showed higher *Nrf2* gene transcription. These data suggest that patients with the *Nrf2* rs35652124 variant, as well as with the B/B genotype in *GST1A1* and [TA]6/6 or [TA]7/7 in the *UGT1A1* gene could benefit more from coffee-mediated effects [145]. Notwithstanding, as we described earlier, although the *Nrf2* rs6721961 and rs35652124 variants are detrimental for antioxidant defense in the setting of inflammatory diseases, rs6706649 has not been associated with cardiovascular diseases [53,55,121]. These data support the potential beneficial effects of coffee consumption in patients carrying these *Nrf2* polymorphisms. Certainly, specific *Nrf2* genetic variations can influence their ability to be activated by bioactive compounds [146]. Coffee consumption exerts a large influence on the Nrf2 pathway by individual genetic variation that might be related to the risk of cardiometabolic diseases.

Another intriguing issue is that gene variants that improve caffeine breakdown are expressed under high or prolonged coffee consumption. GWAS have identified an association between coffee consumption and the *CYP* (*1A1/2*) gene rs2472297 and rs2470893 variants, as well as *AHR* rs4410790 and rs6968865 variants [147,148,149]. It has been established that AHR cooperates with Nrf2 to regulate antioxidant gene expression [150,151,152]. Certainly, AHR senses xenobiotics, including polycyclic aryl hydrocarbons found in roasted coffee, also regulates the transcription of both *CYP* (*1A1/2*) [147]. CYPs enzymes are implicated in xenobiotics metabolism and are essential for caffeine metabolism [142]. Both rs2472297 and rs6968865 are associated with higher CYP activity, which enhances caffeine clearance and allows higher coffee consumption [147]. As we mentioned before, CYP and UGT enzymes are also essential for drug metabolism, including those used in cardiovascular therapy. Therefore, *CYP* and *UGT* polymorphisms might also directly influence pharmacokinetics, contributing to the inter-individual variability.

## 7. Association of Environmental Factors with *Nrf2* and Related Genes Expression in Cardiometabolic Diseases

Environmental pollution includes fine and ultrafine particulate matter (PM). Some of the major components of PM include black carbon, elemental carbon, and organic carbon, which, in turn, might include polycyclic aromatic hydrocarbons, carbonyl compounds, n-alkanes, organic acids, and heterocyclic compounds [153,154]. Environmental pollution has been associated with a higher risk for cardiovascular diseases [155,156]. Since PM causes redox alterations and endothelial dysfunction [157], Nrf2 function seems to be highly relevant. A model of *Nrf2* knock-out mice exposed to PM promoted vascular thickness, increased angiotensin II levels, and worsened cardiac function [158,159]. This phenomenon could also affect patients carrying *Nrf2* polymorphisms that diminish its function. However, we only found one study in patients. Patients carrying the SNPs of *GST* rs1695, *SOD2* rs4880, and *Nrf2* rs1806649 showed higher susceptibility to PM and respiratory impairment due to air pollution [160]. Moreover, in elderly patients with cardiovascular diseases, the exposure of PM such as elemental carbon, black carbon, nitrogen oxides, and, in particular, organic carbon was associated with *Nrf2* gene expression [161].

On the other hand, the gas generated from tobacco smoking contains fine PM and aldehydes, which are implicated in the loss of lung function [162]. Of note, tobacco smoking is also closely related to inflammation and endothelial dysfunction and is the leading factor of atherosclerosis development [163]. In this regard, the *Nrf2* gene variant rs6721961 TT has been found in heavy cigarette smokers [164]. As mentioned earlier in this review, such *Nrf2* gene variant is associated with T2DM prevalence, CAD, coronary atherosclerosis, elevated systolic and diastolic pressure levels, and cardiovascular mortality. Furthermore, a study in a Japanese population found that the *Nrf2* rs6726395 polymorphism is associated with individual susceptibility to loss of lung function, owing to cigarette smoking [165]. Altogether, evidence support these environmental factors, such as air pollution and tobacco smoking, are related to *Nrf2*-related gene function and play a role in the risk of cardiovascular diseases.

## 8. Concluding Remarks

Numerous *Nrf2* polymorphisms have been related to functional alterations associated with cardiometabolic diseases in diverse human populations. SNPs of *Nrf2* and related genes contribute to susceptibility for obesity, inflammation, and diabetes progression, as well as coronary artery disease, hypertension, and cardiovascular mortality. Clearly, the endogenous antioxidant system deficiency is associated with several diseases; hence, the influence of the genetic variation in *Nrf2* and ARE-containing genes that can impair the induction of the Nrf2-driven pathway accounts for susceptibility to suffer cardiometabolic diseases. Polymorphisms of *Nrf2* and related genes have a clear association with the onset and risk of cardiometabolic diseases; therefore, they might be helpful to predict the incidence of cardiovascular events. Aside from their potential application as risk factors markers, *Nrf2*-target gene variants might be modifiers of pharmacokinetics contributing to the inter-individual variability and impairing the effectiveness of cardiovascular drug therapies. A better understanding of the implications regarding the polymorphisms of *Nrf2* and related genes will allow us to design novel cardioprotective strategies to improve clinical outcomes due to complications in responses to therapies among individuals. Although we here describe the influence of *Nrf2* gene variants on cardiovascular diseases, other polymorphisms found in a comprehensive range of genes need to be integrated to further comprehension.

## Figures and Tables

**Table 1:** Association of SNPs of *Nrf2* and related genes with cardiometabolic diseases.

Gene	SNP ID	Study Group	Finding	Reference
*Nrf2*	rs2364723	SAPALDIA cohort 2002/2003 (*N* = 1472) 2010/2011 (*N* = 1235)	Adverse obesity effects on heart rate variability.	[80]
*Nrf2*	rs6721961	Mexican patients: *N* = 627 diabetic subjects and *N* = 1020 controls; Chinese patients: N883 T2DM subjects (383 with diabetic nephropathy).	Associated with T2DM development and complications. Higher risk for CAD and severity of coronary atherosclerosis.	[94,95,108]
*Nrf2*	rs35652124	Indian subjects *N* = 400 (100 with diabetic foot ulcer, 150 T2DM patients, and 150 healthy subjects). *N* = 464 Japanese subjects (285 men and 179 women). N64 healthy African American. *N* = 184 white individuals	Association with insulin resistance and diabetic complications. Cardiovascular disease-associated mortality. Impairment of vasodilation.	[97,104,122]
*Nrf2*	rs2364723	SAPALDIA cohort 2002/2003 (*N* = 1472) 2010/2011 (*N* = 1235). *N* = 1390 subjects from the Vlagtwedde–Vlaardingen cohort. *N* = 809 Chinese volunteers (214 T2DM patients, 236 without diabetic complications, and 359 healthy individuals).	Impairment of cardiovascular function. Low risk of mortality in cardiovascular diseases. Diabetic complications. Associated with lower triglyceride levels.	[59,80,89]
*Nrf2*	rs13001694	*N* = 1390 subjects from the Vlagtwedde–Vlaardingen cohort.	Associated with low risk of mortality in cardiovascular diseases.	[59]
*Nrf2*	rs182428269	*N* = 400 (150 2DM subjects, 150 healthy subjects, and 100 with diabetic complications).	Risk for T2DM development and diabetic complications	[98]
*Nrf2*	rs10497511	*N* = 809 Chinese volunteers (214 T2DM patients, 236 without complications, and 359 healthy individuals).	Diabetic complications.	[89]
*Nrf2*	rs1962142	*N* = 809 Chinese volunteers (214 T2DM patients, 236 without diabetic complications, and 359 healthy individuals).	Diabetic complications.	[89]
*Nrf2*	rs6726395	*N* = 809 Chinese volunteers (214 T2DM patients, 236 without diabetic complications, and 359 healthy individuals).	Diabetic complications.	[89]
*KEAP1*	rs11085735	*N* = 117 patients with fatal and nonfatal cardiovascular events (42 died).	Predictor of cardiovascular events.	[57]
*HMOX1*	rs2364723	*N* = 809 Chinese volunteers (214 T2DM patients, 236 without diabetic complications, and 359 healthy individuals).	Negative associated with diabetic complications.	[89]
*GPx*	rs1050450	416 Mexican women (*N* = 208 healthy subjects, *N* = 208 obese subjects). *N* = 1244 T2DM subjects and *N* = 730 healthy subjects. *N* = 773 Caucasian subjects genotyped from the UCL Diabetes and Cardiovascular disease Study and *N* = 382 Caucasian subjects from the Ealing Diabetes Study. *N* = 396 T2DM patients and *N* = 678 control subjects.	Morbid obesity development, particularly in females. Associated with diabetic neuropathy and development of carotid plaques in patients with diabetes.	[90,91,92,93]
*NQO1*	rs1800566	*N* = 2374 Thai subjects (with and without CAD). *N* = 834 T2DM patients (601 Seoul set and 233 Koyang set). *N* = 130 patients (67 patients with coronary heart disease and 63 healthy individuals).	Associated with severity of coronary atherosclerosis and CAD in females. Controversial association with cardiovascular risk. Associated with hypertension.	[108,116,117]
*CAT*	rs1049982	*N* = 1388 participants > 18 years old (704 women, 300 untreated hypertensive patients).	Risk of hypertension.	[125]
*TXN*	rs2301241	*N* = 1388 participants > 18 years old (704 women, 300 untreated hypertensive patients).	Associated with high blood pressure.	[125]
